# Frequent detection of Saffold cardiovirus in adenoids

**DOI:** 10.1371/journal.pone.0218873

**Published:** 2019-07-03

**Authors:** Kira Lindner, Michael Ludwig, Friedrich Bootz, Ulrike Reber, Zahrasadat Safavieh, Anna Maria Eis-Hübinger, Stephan Herberhold

**Affiliations:** 1 ENT Department, Head and Neck Surgery, University of Bonn, Bonn, Germany; 2 Department of Clinical Chemistry and Clinical Pharmacology, University of Bonn, Bonn, Germany; 3 Institute of Virology, University of Bonn Medical Centre, Bonn, Germany; Arizona State University Biodesign Institute, UNITED STATES

## Abstract

Saffold virus (SAFV) is classified into the *Cardiovirus* genus of the *Picornaviridae* family. Up to now, eleven genotypes have been identified however, their clinical significance remains unclear. Here, we investigated the presence of SAFV in asymptomatic patients admitted for adenoidectomy. A total of 70 adenoid tissue samples were collected from children with clinical symptoms caused by hypertrophy of adenoids but without symptoms of airway infection. Samples were investigated for SAFV by RT-nested PCR and sequence analysis. Eleven of 70 (15.7%) samples were positive for SAFV. Nasopharyngeal swabs were available from 45 children just before surgery. SAFV was rarely found and only in children with SAFV-positive adenoids 2/8. Our findings indicate that the presence of SAFV seems to be more frequent in adenoid tissue than expected. This could support the notion of a longer than previously anticipated persistence of SAFV nucleic acids in the respiratory tract and possibly a chronic infection. Further investigations are necessary to establish the role of SAFV infection in humans.

## Introduction

Saffold virus (SAFV) is an emerging pathogen identified in the United States in 2007 [1]. The virus´ name traces back to the name of the first author of the original description. The virus is classified in the species Cardiovirus B (formerly named Theilovirus) within the genus *Cardiovirus* (genus supergroup 1) of the family *Picornavirida*e [1–4].

SAFV is closely related to the other members of the Cardiovirus B species, i.e., Theiler's murine encephalomyelitis virus (TMEV), Theiler-like rat virus (Thera virus, TRV), and Vilyuisk human encephalomyelitis virus (VHEV). SAFV is a small non-enveloped virus with a single-stranded RNA of about 8,050 nucleotides. Up to now, eleven different SAFV genotypes have been identified (http://www.picornaviridae.com/).

SAFV-1 has initially been isolated from a fecal specimen of an 8-month-old girl with fever of unknown origin [1]. Following this observation, SAFV was detected frequently in nasal specimens of children with respiratory infections (0.2%-24%, [5]) and stool samples from children suffering from acute gastroenteritis (0.2%-3%, [5]) [1, 4–13]. Due to these findings, SAFV was supposed to be a relevant new human pathogen, especially in children. According to antibody seroprevalence studies, SAFV infection is highly common and occurs early in life, with approximately 80% of seropositive children at the age of 2 years [14–16]. SAFV is spread worldwide [1, 3–4, 6, 9–10, 12–17].

The association of SAFV with various diseases is currently under research. Besides respiratory and gastrointestinal illnesses, the disease spectrum may involve type 1 diabetes and neurological disorders [7, 17]. Furthermore, the virus was detected in nasopharyngeal swabs from children with exudative tonsillitis (9/37, [18]) and in autopsy samples from myocardium, lung and blood from a child with myocarditis [19]. Additionally, the virus was found in feces from Asian children with non-polio acute flaccid paralysis [9] and in cerebrospinal fluid specimens of patients with aseptic meningitis [12, 20–21]. This is of particular importance, because other members of the Cardiovirus B species are neurotropic. E. g., TMEV is known to cause a multiple sclerosis (MS)-like syndrome in mice [21]. However, there are also reports i) describing SAFV detection with similar percentages in healthy and diseased individuals, e.g., in stool samples [9, 22–23] and, ii) reports failing to detect SAFV in samples from diseased patients, e.g., in cerebrospinal fluid from individuals with aseptic meningitis, encephalitis, and MS [24–25], and, finally, iii) reports co-detecting SAFV with common gastroenteritis pathogens in stool samples in case of diarrhea [22]. The true clinical significance and pathophysiology of SAFV thus has to be elucidated.

In order to extend our knowledge on SAFV presence in healthy children, we analyzed adenoid tissue and throat swab samples from children who did not display symptoms of a respiratory tract infection for SAFV RNA. Samples were collected in the course of elective adenoidectomy.

## Materials and methods

### Patients and ethical approval

A total of 70 children (41 males and 29 females), ranging from 0.8 to 12 years of age, were included in the prospective study. The median age at adenoidectomy was 3 years (IQR 2.5 years). All parents gave written informed consent. The study was approved by the ethics committee of the University of Bonn (044/11) in written form.

### Specimen collection

From December 2014 to May 2015, adenoid tissue samples were obtained from the 70 patients consecutively admitted for adenoidectomy at the Bonn University Medical Centre, Department of Otorhinolaryngology. Ear, nose and throat specialists determined the indication for surgery. From 45 of these patients, a throat swab was taken just before the surgical procedure. All patients had clinical symptoms caused by hypertrophy of adenoids. At the time of surgery and the 2 weeks before, no children displayed symptoms of acute upper or lower airway infection.

### RNA extraction, RT-nested PCR and sequence analysis

Extirpated adenoids were picked up in the surgical room and transported on ice to the laboratory for immediate preparation. For nucleic acid preparation, approximately 25 mg of adenoid tissue was crushed mechanically with a scalpel followed by incubation with 600 μL RLT buffer (Qiagen Hilden, Germany) and 1% β-mercaptoethanol (Sigma-Aldrich/Merck, Munich, Germany). The lysate was homogenized by using QIAshredder homogenizer spin columns (Qiagen) according to manufacturer´s instructions. After addition of 1 volume 70% ethanol to the homogenized lysate, RNA was extracted from the sample with the RNeasy Mini Kit (Qiagen) according to the manufacturer´s protocol. All precautions to avoid contamination were strictly adhered to.

SAFV RNA was detected by real-time reverse transcription (RT-)PCR using the primers and probe as previously described [12], with sequences as follows: CF723: TGTAGCGACCTCACAGTAGCA; CR888: CAGGACATTCTTGGCTTCTCTA; CP797: FAM-AGATCCACTGCTGTGAGCGGTGCAA-BHQ1. RT-PCR was performed in a volume of 25 μL containing 5 μL RNA preparation (approximately 1.25 mg) and by using SuperScriptIII One-Step RT-PCR System with Platinum *Taq* DNA Polymerase (Invitrogen/ThermoFisher Scientific, Schwerte, Germany) and 1 μg bovine serum albumin (VWR International, Langenfeld, Germany). RT and cycling conditions were 52°C for 20 min, denaturation at 94°C for 3 min, followed by 45 PCR cycles, each consisting of 95°C for 15 sec and 58°C for 30 sec. The PCR amplified a 187-bp fragment of the SAFV genome within the 5´ untranslated region (5´UTR). The limit of detection 95% (LOD_95_) was 9 copies per reaction.

Samples testing positive by RT-PCR were subjected to nested RT-PCR for amplifying a larger genomic stretch, with an inner fragment of approximately 592 bp within the 5´ untranslated region (nucleotide positions [nts] 204–795, according to GenBank number EF165067; without primers, nts 224–775; please note the small SAFV strain-specific differences in fragment length) followed by nucleotide sequencing. SuperScript III One-Step RT-PCR System with Platinum *Taq* DNA Polymerase and 1 μg BSA was used for first round of amplification and Invitrogen Platinum *Taq* DNA Polymerase was used for second round amplification. Reaction volume was 25 and 50 μL in the first and second round, respectively, each with 5 μL target. RT and amplification primers were as follows: first round, Cardio-Universal-F1, 5´-GCTAATCAGAGGAAAGTCAGCATT-3´; Cardio-Universal-R1, 5´- GACCACTTGGTTTGGAGAAGCT-3´; second round, Cardio-Universal-F2, 5´-CAGCATTTTCCGGCCCAGGC-3´, Cardio-Universal-R2, 5´-ATCCACGGGGCTTTTGGCCG-3´. RT and cycling conditions were as follows: RT and first round, 48°C for 30 min, 95°C for 5 min, 35 cycles each consisting of 95°C for 1 min, 50°C for 1 min, 72°C for 1 min, followed by 72°C for 5 min; nested PCR, 95°C for 3 min, 35 cycles (94°C for 1 min, 60°C for 1 min, 72°C for 1 min), 72°C for 5 min. Amplified products were visualized on agarose gels and were subjected to DNA cycle sequencing using BigDye Terminator technology (3130XL Genetic Analyzer, Applied Biosystems, Foster City, USA). Sequencing was done in both directions. Sequences were manually reviewed and compared with genome sequences in GenBank.

Viral RNA was quantified by use of an *in vitro* transcript of a plasmid-based standard (pCR4.0 TOPO-TA vector, ThermoFisher) derived from an 803-bp PCR amplicon encompassing the screening real-time PCR target region.

Throat swabs were taken by flocked swabs (Copan) and were dissolved in 500 μL phosphate-buffered saline. Viral nucleic acid was prepared by use of the QIAamp Viral RNA Mini Kit (Qiagen) and eluted in 100 μL. Testing for SAFV RNA was performed by real-time RT-PCR as mentioned above.

Testing for typical respiratory viruses was performed by RT-PCR as described previously [26]. Tested viruses were Influenza A and B viruses, Human parainfluenza viruses 1–4 (now termed Human respiroviruses 1 and 3, Human rubulaviruses 2 and 4), Human Rhinovirus, Human respiratory syncytial virus, Human metapneumovirus, Enterovirus, Human parechovirus and Human coronaviruses 229E, NL63, HKU-1, and OC43, and Human adenovirus.

To strengthen our findings, we subsequently tested our SAFV-positive tissues and swabs for the non-respiratory viruses Norovirus and Zika virus by use of the RealStar Norovirus RT-PCR Kit 1.0 (Altona Diagnostics) and the RealStar Zika Virus RT-PCR Kit 3.0 (Altona Diagnostics) according to the manufacturer´s protocols.

## Results

Out of 70 adenoid tissue specimens tested by the screening RT-PCR, eleven (15.7%) were positive for SAFV RNA (Table 1). SAFV-positive cases were from both, males (7/41, 17.1%) and females (4/29, 13.8%). The ages of the SAFV-positive patients ranged from 3 to 10 years (median 5 years). The detailed age distribution of SAFV positives and SAFV negatives is given in Table 2.

**Table 1:** Characteristics of children with SAFV positive adenoids.

Patient	Gender, age (yrs)	SAFV copies/rx in		Other viruses in	
		adenoid tissues	throat swabs	adenoid tissues	throat swabs
1	m, 3	4.39 log_10_	n.d.	EV, HPIV-3, HBoV	n.d.
2	m, 3	4.38 log_10_	n.d.	HBoV, RSV, RV	n.d.
3	m, 3	2.70 log_10_	3.04 log_10_	EV, HBoV, HPIV-2, -4, RSV, RV	RV
4	f, 4	2.69 log_10_	neg.	HAdV, HBoV, HCoV HKU-1, HBoV, RV	HAdV
5	f, 4	2.04 log_10_	neg.	EV	RV
6	f, 5	~ 1 log_10_	neg.	EV, HPeV	FLUAV
7	m, 5	1.62 log_10_	neg.	EV, HPeV, HPIV-3	neg.
8	m, 5	1.66 log_10_	neg.	EV, HAdV, HBoV, HCoV OC43, HPIV-2	HAdV
9	f, 6	2.94 log_10_	2.56 log_10_	EV, HBoV, HPIV-1, -2	HAdV, HBoV
10	m, 8	~1 log_10_	n.d.	EV, HBoV	n.d.
11	m, 10	3.65 log_10_	neg.	EV	neg.

yrs, years; m, male; f, female; rx, reaction

SAFV, Saffold cardiovirus; n.d., not done; neg., negative; EV, Enterovirus; HPIV, Human parainfluenza virus; HBoV, Human bocavirus; RV, Rhinovirus; RSV, Human respiratory syncytial virus; HCoV, Human coronavirus; HPeV, Human parechovirus; HAdV, Human adenovirus; FLUAV, Influenza A virus.

**Table 2:** Age distribution of patients with SAFV or other viruses in adenoid tissues.

Age (yrs)	No. tested children	No. SAFV-positives	No. SAFV-negatives	No. of children with other viruses		
				0 virus	1–2 viruses	> 2 viruses
0–1	5	0	5	0	2	3 (60%)
2–3	32	4	28	0	5	27 (84%)
4–5	17	4	13	1	4	12 (71%)
6–7	8	1	7	0	3	5 (63%)
8–9	2	1	1	0	2	0
10–11	5	1	4	0	5	0
12–13	1	0	1	1	0	0
Total (%)	70	11 (16%)	59 (84%)	2 (3%)	21 (30%)	47 (67%)

yrs, years; No., number; SAFV, Saffold cardiovirus.

SAFV RNA concentration of the positive tissues ranged between <10 and 1x 10^4^ copies/reaction. Three specimens showed values over 1,000 copies/reaction.

In every case of the 11 SAFV-positive adenoid samples, classical respiratory viruses were also found (Table 1), with Enterovirus being the most frequent one (n = 9, 81.8%) followed by Human bocavirus (n = 7, 63.6%) and one of the four Human parainfluenza viruses (n = 5; 45%). SAFV was also detected in combination with Human rhinovirus (n = 2), Human parechovirus (n = 2), Human adenovirus (n = 2), Human respiratory syncytial virus (n = 2), and Human coronaviruses HKU-1 and OC43 (n = 1 each). The number of co-detected viruses ranged between one and six. The non-respiratory viruses Norovirus and Zika virus were not found.

From 45 of the 70 patients tested, a throat swab was collected just before surgery. Thereof, eight throat swabs were from individuals with SAFV-positive adenoids. Two of 45 swabs tested positive for SAFV RNA (Table 1). These two swabs were derived from individuals with SAFV-positive adenoid tissue indicating a rate of 2/8 (25%). Viral load in the swabs was rather low, i. e., 4.5x 10^2^ and 1.1x 10^2^ copies/mL. In both swabs, classical respiratory viruses were also found (case #1, Human rhinovirus; case #2, Human adenovirus and Human bocavirus). None of the throat swabs derived from children with SAFV RNA-negative adenoid tissues tested positive.

Fifteen of the 45 (33%) swabs were negative for any of the tested viruses while 30 yielded at least one virus, albeit at low concentration (Table 3). Most frequently, Human bocavirus was found (10/30 = 33%), followed by Human rhinovirus and Human adenovirus (9/30 each = 30%), Enterovirus (6/30 = 20%), Human parainfluenza viruses (4/30 = 13%), Human coronaviruses (3/30 = 10%), Human parechovirus and Influenza virus A (1/30 = 3% each).

**Table 3:** Age distribution of patients with SAFV or other viruses in throat swabs.

Age (yrs)	No. tested Children	No. SAFV-positives	No. SAFV-negatives	No. of children with other viruses		
				0 virus	1–2 viruses	> 2 viruses
0–1	2	0	2	0	1	1
2–3	21	1	20	4	15	2
4–5	12	0	12	6	6	
6–7	6	1	5	2	4	
8–9	0					
10–11	4	0	4	3	1	
12–13	0					
Total (%)	45	2 (4%)	43 (96%)	15 (33%)	27 (60%)	3 (7%)

yrs, years; No., number; SAFV, Saffold cardiovirus.

Partial genome sequencing was only successful in three of the SAFV-positive adenoid tissue samples. BLAST alignment of the analyzed PCR product revealed a nearly complete match with the sequences of SAFV2 strains, deposited in the database. Hence, SAFV2 turned out to be the most likely candidate. In the remaining samples, amplification was hampered, most probably because of low viral load.

## Discussion

To the best of our knowledge, this is the first study demonstrating SAFV in adenoid tissues. Out of 70 adenoid tissue specimens from children, 11 (15.7%) were positive for SAFV. Noteworthy, all children underwent elective adenoidectomy and did not display symptoms indicative of an infection of the upper or lower respiratory tract at time or within two weeks preceding surgery. Although the number of SAFV RNA-positive throat swabs was low, SAFV was only detectable in throat swabs from children whose adenoids also contained SAFV RNA.

In literature, there are only very few reports describing analysis of SAFV in specimens from asymptomatic individuals. Zhang and colleagues [27] collected 352 throat swabs from asymptomatic children, aged 4 to 78 months. In contrast to our results, these authors did not detect any SAFV positives among the asymptomatic children while 25/1829 (1.37%) swabs from children with respiratory symptoms were positive. The authors applied the same RT-PCR protocol as we did. Except for that particular study sampling oropharyngeal specimens, as we did, all other information on healthy children is derived from analysis of feces. Drexler and colleagues [12] did not detect SAFV-RNA in stool samples from 39 control children while the detection rate in children with diarrhea was 0.7% (6/844). In contrast to these two studies [12, 27] reporting absence of SAFV in asymptomatic children, there are three reports describing a similar or an even higher prevalence of SAFV in feces from healthy children compared to children with gastrointestinal illness (2.8% *vs*. 1.5% [22]; 2.8% *vs*. 2.6% [23]) or nonpolio acute flaccid paralysis (12.2% *vs*. 8.8% [9]). Our results demonstrating presence of SAFV in non-diseased individuals might be consistent with the results of these three studies, although the body site where the specimens were collected was quite different.

Surprisingly, detection of SAFV seems to be more frequent in adenoid tissue (15.7%) than in non-tissue specimens from the respiratory tract. In a recent study, SAFV was detected in 9.7% of throat swabs from 226 Taiwanese-children with respiratory symptoms [4]. In a larger cohort of 1,525 children with acute respiratory infections in Japan, SAFV2 was found in 3.5% of nasopharyngeal swabs [28]. In another study from Bejing including 1,558 children, SAFV was detected in 0.6% of nasopharyngeal aspirates from 506 patients with upper respiratory tract infection and in 0.4% of 1,032 patients with lower respiratory tract infections [29]. The hypothesis of a higher SAFV prevalence in adenoid tissue than in non-tissue specimens from the respiratory tract might be underpinned by our results detecting SAFV in 15.7% of adenoid tissue samples vs. 4.4% in throat swabs, although the difference was not statistical significant (*p =* 0.059), most probably due to the low numbers analyzed. However, there is one study reporting a rather higher rate of SAFV-positive nasopharyngeal swabs (9/37 = 33.3%) taken from children with exudative tonsillitis [18] than we found in the adenoid tissue.

Intriguingly, in our study testing healthy children, SAFV was only detected in throat swabs from individuals also positive for SAFV in their adenoids (2/8). Noteworthy, viral concentration in the swabs was rather low. However, comparison with results from other studies is not possible due to lack of information in literature about SAFV RNA load.

In our study, three samples could probably belong to SAFV2. According to current knowledge, genotype 2 appears to be the most prevalent one in Central Europe. In a large study performed in Denmark, all 38 SAFV-positive feces (2.8%) belonged to that type [17].

Besides SAFV, a high rate of common respiratory viruses was detected in every case of the SAFV-positive adenoid tissue sample. Enterovirus and Human parechovirus deserve a special mention regarding their belonging to the *Picornaviridae* family, as does SAFV. Overall, Enterovirus was the most frequent co-detected virus (9/11 (81.8%)), followed by Human bocavirus (7/11 (63.6%)). In the SAFV-negative tissues, both viruses were less frequently found, i.e., Enterovirus in 31/59 (52.54%) and Human bocavirus in 34/59 (57.62%) samples. However, the differences between the SAFV-positive and negative group are not statistically significant. Notwithstanding, the high prevalence of various viruses in adenoid tissue is surprising. One may speculate as to whether children with adenoid hypertrophy requiring adenoidectomy underwent a greater number of infections resulting in a higher rate of tissue-associated viral nucleic acid than children without adenoid hypertrophy. Alternatively, storage of viruses or viral genomes in adenoids might be a general phenomenon in childhood. The finding of DNA and RNA of multiple viruses, at least of fragments, supports the notion of a longer than previously anticipated persistence of viral nucleic acids in adenoids. Sato et al. [30] speculated on a normal viral flora and a chronicity of selected respiratory viruses. Alternatively, viral presence in adenoid tissue might play a role with respect to the immune response. Interestingly, in contrast to adenoid tissue (Table 2), we rarely found nucleic acid from multiple viruses (> 2) in nasopharyngeal swabs (Table 3).

Lin et al. conducted a study which supports the hypothesis that SAFV might play a role in the pathogenesis of upper respiratory infections. Yet, the authors critically noted that it would need to be checked by looking for the virus in asymptomatic patients in further studies [4]. Although our study focused on asymptomatic individuals, the results of our study do not contribute to the answer of the question about the true clinical significance of SAFV detection. However, our study at least demonstrated that SAFV, and in addition a spectrum of other respiratory viruses, can be detected at a relatively high rate in the adenoids of asymptomatic children. Additional studies with enlarged sample size including both adolescents and adults as well as attempts for SAFV isolation in cell culture are needed to clarify the duration period of SAFV persistence in tissue as well as the virus´ ability for replication in asymptomatic individuals.

The GenBank accession numbers for the three SAFV nucleotide sequences were as follows: MK182597, MK182598, and MK182599.

## Supporting information

S1 Table Lindner-et-al-Table I-1 (complete specimen collection). (DOC)

S2 Table Lindner-et-al-Table II extendet (characteristics of patients with positive adenoid tissue). (DOC)

S3 Table Cardio-PCR. (DOC)

S4 Table Cardio Sequenzier-PCR. (DOC)

S5 Table Cardio Rohdaten. (ZIP)
