# Supplementary material for: Frequent detection of Saffold cardiovirus in adenoids
Source: PLoS One. 2019 Jul 3;14(7):e0218873. doi: 10.1371/journal.pone.0218873 (PMC6608973; doi:10.1371/journal.pone.0218873)
Supplement: S1 Table — (DOC) [file pone.0218873.s001.doc]

Table I. Complete specimen collection

| No. | Gen-der | Age  (yrs) | Detected virus in tissue (CT) | Detected virus in throat swab (CT) |
| --- | --- | --- | --- | --- |
| 1 | m | 3 | Parainfluenza-1 (40,00), Parainfluenza-3 (40,00), Adenovrus (35,13), Enterovirus (40,0), Bocavirus (37,68) | no throat swab |
| 2 | m | 8 | **Cardiovirus (42**), Enterovirus (36,25), Bocavirus (>40) | no throat swab |
| 3 | m | 3 | Entero (32,79), Parainfluenza-1 (40,00), Parainfluenza-3 (39,62), Bocavirus (30,11) | no throat swab |
| 4 | f | 2 | Enterovirus (40,00), Respiratory syncytialvirus (40,00), Parainfluenza-3 (40,00), Rhinovirus (36,91), Influenza A (23,99), Bocavirus (30,75) | Bocavirus (41) |
| 5 | f | 2 | Enterovirus (30,35), Parainfluenza-3 (40,00), Adenovirus (36,30), Human coronavirus OC43 (37,08), Influenza A (23,94), Bocavirus (25,78) | Enterovirus (32,52) |
| 6 | m | 1 | Enterovirus (33,76), Parainfluenza-4 (33,22), Parainfuenza-2 (40,00), Respiratory syncytialvirus (35,61), Parainflunza-3 (35,58), Human coronavius NL63 (30,92), Adenovirus (34,91), Human coronavirus OC43 (30,82), Bocavirus (28,81) | Respiratory syncytialvirus (33,32), Parainluenza-3 (40,00), Human cornavirus NL63 (38,66), Adenovirus (35,17), Human coronavirus OC43 (36,76), Bocavirus (35,76) |
| 7 | f | 5 | **Cardiovirus (41,94),** Enterovirus (35,29), Parechovirus (33,99) | Influenza A (33,72) |
| 8 | m | 4 | Enterovirus (36,19), Human coronavirus NL63 (32,83), Adenovirus (36,51) | Adenovirus (38,98) |
| 9 | m | 4 | Enterovirus (33,74), Parainfluenza-3 (38,46), Human cornavirus NL63 (31,97), Adenovirus (39,36) | no virus found |
| 10 | m | 5 | **Cardiovirus (38,62),** Enterovirus (38,89), Parainfluenza-3 (40,00), Parechovirus (39,16) | no virus found |
| 11 | m | 1 | Enterovirus (35,99), Parainfluenza-3 (33,73), Adenovirus (40,00), Parechovirus (39,25), Bocavirus (33,24) | Adenovirus (28,76), Bocavirus (32,72) |
| 12 | f | 6 | Adenovirus (40,00) | Influenza A (39,38) |
| 13 | f | 2 | Enterovirus (40,00), Rhino virus (40,00), Human coronavirus OC43 (33,92), Influenza A (35,18), Bocavirus (33,03) | Rhinovirus (40,00) |
| 14 | f | 2 | Enterovirus (30,45), Parainfluenza-2 (38,42), Human coronavirus NL63 (33,81), Human coronavirus OC43 (35,17), Influenza A (29,53), Bocavirus (24,47) | Rhinovirus (36,12), Bocavirus (34,84) |
| 15 | m | 4 | Enterovirus (37,40) | Adenovirus (37,87) |
| 16 | m | 10 | **Cardiovius (32,41),** Enterovirus (40,00) | no virus found |
| 17 | m | 2 | Enterovirus (40,00), Parainfluenza-2 (40,00), Bocavirus (27,98) | Enterovirus (40.00), Bocavirus (32,05) |
| 18 | m | 3 | Parainfluenza-3 (40,00), Adenovirus (36,61), Bocavirus (>40) | Rhinovirus (31,49) |
| 19 | m | 4 | Parainflueza-3 (38,55) Rhinovirus (34,97), Human coronavirus OC43 (37,10), Bocavirus (>40) | no virus found |
| 20 | m | 3 | Enterovirus (40,00), Human coronavirus NL63 (22,71), Bocavirus (33,02) | no throat swab |
| 21 | f | 1 | Parainfuenza-3 (40,00), Bocavirus (26,49) | no throat swab |
| 22 | m | 3 | Respiratory syncytialvirus (40,00), Parainfluenza-3 (40,0), Influenza A (29,51), Bocavirus (31,25) | no throat swab |
| 23 | f | 5 | Enterovirus (40,00) | no throat swab |
| 24 | m | 2 | Enterovirus (33,92), Adenovirus (40,00), Bocavirus (>40) | no throat swab |
| 25 | f | 3 | Rhinovirus (33.71), Human coronavirus NL63 (23,73) | no throat swab |
| 26 | f | 4 | Rhinovirus (31,98), Parechovirus (40,00), Influenza B (29,86) | no throat swab |
| 27 | f | 5 | Enterovirus (36,80), Parainfluenza-2(39,27) Respiratory syncytialvirus (40,00), Rhinovirus (28,11), Parechovirus (40,00) Influenza A (34,14), Influenza B (27,44), Bocavirus (>40) | no throat swab |
| 28 | f | 6 | Parainfluenza-2 (40,0), Rhinovirus (35,7) | no throat swab |
| 29 | m | 9 | Adenovirus (35,05) | no throat swab |
| 30 | f | 2 | Enterovirus (39,34), Respiratory syncytialvirus (37,63), Parainfluenza-3 (35,49), Adenovirus (34,51), Bocavirus (32,47) | no throat swab |
| 31 | f | 1 | Enterovirus(40,00), Parainfluenza-3(36,53), Human coronavirus NL63(37,15), Influenza A(30,62), Bocavirus (30,00) | no throat swab |
| 32 | m | 3 | Enterovirus (40,0), Adenovirus (39,41), Parechovirus (39,52) | no throat swab |
| 33 | f | 12 | no virus found | no throat swab |
| 34 | m | 5 | Adenovirus (33,78) | no throat swab |
| 35 | m | 3 | **Cardiovirus SAFV2 (29,41),** Enterovirus (40,00), Parainfluenza-3 (40,00), Bocavirus (29,98) | no throat swab |
| 36 | m | 3 | **Cardiovirus SAFV2 (29,36),** Rhinovirus (38,14), Respiratory syncytial virus (40,0), Bocavirus (36,87) | no throat swab |
| 37 | m | 2 | Adenovirus (40,00), Human coronavirus OC43 (36,93), Bocavirus (39,40) | no throat swab |
| 38 | f | 6 | Enterovirus (40,00), Rhinovirus (36,58), Adenovirus (40,00), Bocavirus (37,43) | no throat swab |
| 39 | m | 10 | Parainfluenza-3 (36,25), Adenovirus (40,00) | no throat swab |
| 40 | m | 4 | Enterovirus (30,34), Parainfluenza-1 (40,00), Parainfluenza-2 (40,00), Rhinovirus (33,66), Adenovirus (38,20), Bocavirus (37,73) | Enterovirus (37,12)  Rhinovirus (33,21) |
| 41 | m | 2 | Parainfluenza-3 (37,76), Bocavirus (32,47) | Bocavirus (38) |
| 42 | f | 5 | no virus found | no virus found |
| 43 | m | 3 | **Cardiovirus SAFV2 (34,54),** Enterovirus (39,00), Parainfluenza-2 (35,22), Respiratory syncytialvirus (40,00), Rhinovirus (36,58), Bocavirus (37,36) | Cardiovirus (33,17)  Rhinovirus (35,60) |
| 44 | m | 5 | Parainfluza-3 (40,00), Human coronavirus NL63 (36,72), Adenovirus (40,00) | no throat swab |
| 45 | m | 3 | Enterovirus (40,00),Parainflueza-3 (40,00), Rhinovirus (36,11), Bocavirus (37,87) | no virus found |
| 46 | f | 2 | Enterovirus (29,67), Parainfluena-3 (36,22), Parechovirus (37,11), Bocavirus (33,12) | Enterovirus (32,74), Parechovirus (40,00), Bocavirus (36,05) |
| 47 | f | 4 | **Cardioirus (34,91),** Rhinovirus (32,94), Human coronavirus HKU-1 (40,00), Adenovirus (33,34), Bocavirus (32,77) | Adeno (38,09) |
| 48 | f | 2 | Enterovirus (28,62), Adenovirus (35,40), Parechovirus (36,18), Bocavirus (33,62) | Enterovirus (29,67) |
| 49 | m | 7 | Enterovirus (33,24), Parainfluenza-2 (40,00), Respiratory syncytialvirus (40,00), Rhinovirus (37,93) | Enterovirus(36,70) |
| 50 | m | 3 | Parainfluenza-3 (34,60), Bocavirus (25,01) | no virus found |
| 51 | m | 11 | Enterovirus (40,00), Human coronavirus OC43 (37,62) | no virus found |
| 52 | f | 6 | **Cardiovirus (32,33),** Enterovirus (35,92), Parainfluenza-1 (38,58),-Parainfluenza-2 (37,42), Bocavirus (33,03) | Cardiovirus (35,50)  Adenovirus (38,39), Bocavirus (40,0) |
| 53 | f | 3 | **Cardiovirus (42,52),** Enterovirus (40,00) | Rhinovirus (36,69) |
| 54 | f | 5 | Parainluenza-2 (40,00), Adenovirus (39,30), Human coronavius OC43 (38,81), Bocavirus (34,02) | no virus found |
| 55 | f | 10 | Parainfuenza-2 (40,00), Rhinovirus (37,24) | no virus found |
| 56 | m | 2 | Enterovirus (32,55), Rhinovirus (31,49), Adenovirus (35,09), Bocairus (30,33) | Rhinovirus (35,48) |
| 57 | m | 0 | Rhinovirus (36,11), Adenovirus (35,02) | no throat swab |
| 58 | m | 5 | Enterovirus (40,00), Rhinovirus (38,15), Influenza B (31,48), Bocavirus (38,00) | no virus found |
| 59 | m | 6 | Adenovirus (37,44), Human coronavirus OC43 (37,18) | no virus found |
| 60 | m | 11 | Rhinovirus (38,25), Adenovirus (38,33) | Adenovirus (40,0) |
| 61 | m | 6 | Parainfluenza-2 (40,00), Humancoronavirus NL63(37,66), Adenovirus (34,60) | no virus found |
| 62 | m | 3 | Rhinovirus (29,19), Human coronavirus NL63 (32,42), Adenovirus (35,05), Parainfluenza-1 (40,00), Bocavirus (30,30) | Rhinovirus (24,78), Human coronavirus NL63 (36,97), Bocavirus (35,94) |
| 63 | f | 3 | Parainfuenza-2 (37,96), Respiratory syncytialvirus (37,59), Parainfluenza-3 (29,31), Bocavirus (25,82) | Parainfluenza-3 (34,09), Bocavirus (32,79) |
| 64 | f | 3 | Enterovirus (37,44), Parainfluenza-2 (39,57), Bocavirus (34,78) | Adenovirus (33,91) |
| 65 | f | 3 | Parainfluenza-3 (37,69), Humancoronavirus OC43 (37,61) | no virus found |
| 66 | m | 6 | Parainfluenza-2 (40,00), Human coronavirus OC43 (37,02), Bocavirus (>40) | Parainflueza-2 (40,0), Human coronavirus OC43 (37,19) |
| 67 | f | 3 | Parainfluenza-3 (37,78), Human coroavirus NL63 (36,83), Bocavirus (33,32) | Rhinovirus (27,66) |
| 68 | f | 3 | Enterovirus (40,00), RSV (38,19), Parainfluenza-3 (40,00), Human coronavirus OC37 (37,89), Bocavirus (31,87) | No virus found |
| 69 | m | 5 | **Cardiovirus (39,87),** Enterovirus (40,00), Parainfluenza-2 (40,00), Adenovirus (40,00), Human coronavirus OC43 (35,83), Bocavirus (>40) | Adenovirus (40,00) |
| 70 | m | 3 | Parainfluenza-1 (35,10), Parainfluenza-2 (40,00), Parechovirus (40,00), Rhinovirus (38,83) | Parainfluenza-1 (31,87) |

f, female; m, male; CT, cycle threshold.
