# Supplementary material for: Frequent detection of Saffold cardiovirus in adenoids
Source: PLoS One. 2019 Jul 3;14(7):e0218873. doi: 10.1371/journal.pone.0218873 (PMC6608973; doi:10.1371/journal.pone.0218873)
Supplement: S2 Table — (DOC) [file pone.0218873.s002.doc]

| Patient | Gender and age (yrs) | Tissue samples  Cardiovirus copies/1,25 mg | Throat swabs Cardiovirus  copies/ml | Co-infecting virus tissue (CT) | Co-infecting virus swab (CT) |
| --- | --- | --- | --- | --- | --- |
| 1 | m,8 | 42 1,22 copies/run=1,25 mg | no throat swab | Enterovirus (36,25), Bocavirus (>40) | no throat swab |
| 2 | f,5 | 41,94 1,22 | negative | Enterovirus (35,29), Parechovirus (33,99) | Influenza A(33,72) |
| 3 | m,5 | 38,62 9,78 | negative | Enterovirus(38,89), Parainfluenza-3 (40,00), Parechovirus (39,16) | no virus found |
| 4 | m,10 | 32,41 1251,84 | negative | Enterovirus (40,00) | No virus found |
| 5 | m,3 | 29,41; SAFV2 10014,72 | no throat swab | Enterovirus (40,00), Parainfluenza-3 (40,00), Bocavirus (29,98) | no throat swab |
| 6 | m,3 | 29,36; SAFV2 10014,72 | no throat swab | Rhinovirus (38,14), Respiratory syncytialvirus (40,00), Bocavirus (36,87) | no throat swab |
| 7 | m,3 | 34,54; SAFV2 156,48 | 33,17  4,5x10^2^ | Enterovirus (39,00), Parainfluenza-2 (35,22),-4 (40,00), Respiratory syncytialvirus (40,00), Rhinovirus (36,58), Bocavirus (37,36) | Cardiovirus (33,17), Rhinovirus (35,60) |
| 8 | f,4 | 34,91 156,48 | negative | Rhinovirus (32,94), Human coronavirus HKU-1 (40,00), Adenovirus (33,34), Bocavirus (32,77) | Adenovirus (38,09) |
| 9 | f,6 | 32,33 1251,84 | 35,5  1,1x10^2^ | Enterovirus (35,92), Parainfluenza-1 (38,58),-2 (37,42), Bocavirus (33,03) | Cardiovirus (35,5), Adenovirus (38,39), Bocavirus (40,0) |
| 10 | f,4 | 42,52 1,22 | negative | Enterovirus (40,00) | Rhinovirus (36,69) |
| 11 | m,5 | 39,87 4,89 | negative | Enterovirus (40,00), Parainfluenza-2 (40,00), Adenovirus (40,00), Human coronavirus OC43 (35,83), Bocavirus (>40) | Adenovirus (40,00) |

Table II. Characteristics of patients with positive adenoid tissues

f, female; m, male; CT, cycle threshold.
