# Supplementary material for: Frequent detection of Saffold cardiovirus in adenoids
Source: PLoS One. 2019 Jul 3;14(7):e0218873. doi: 10.1371/journal.pone.0218873 (PMC6608973; doi:10.1371/journal.pone.0218873)
Supplement: S3 Table — (DOC) [file pone.0218873.s003.doc]

**Cardiovirus real-time PCR**

3 µl Aqua dest

12,5 µl 2x Reaktions-Mix, Invitrogen SSC III / Platinum Taq - OneStep RT-PCR

1 µl CF-723 (Stock: 10µM)

1 µl CR-888 (Stock: 10µM)

0,5 µl CP-797 (Stock: 10µM)

1 µl BSA (1 mg/ml), Roche

1 µl SSC III / Platinum Taq, Invitrogen

20 µl + 5 µl RNA template (Qiagen Viral RNA mini kit)

Reagents

Invitrogen SuperScript III OneStep RT-PCR System mit Platinum Taq, #12574-026

Roche BSA (20 mg/ml), #10711454001

Qiagen Viral RNA mini kit; #52904

LightCycler, cycling conditions

52°C 20 min

94°C 3 min

45 cycles:

95°C 15 sec

58°C 30 sec; single read step, F1 (530 nm)

Primer/Probe sequences

CF723: TGT AGC GAC CTC ACA GTA GCA

CR888: CAG GAC ATT CTT GGC TTC TCT A

CP797: FAM-AGA TCC ACT GCT GTG AGC GGT GCA A-BHQ1

Drexler et. al. Emerging Inf. Dis. (2008)
