# Supplementary material for: Frequent detection of Saffold cardiovirus in adenoids
Source: PLoS One. 2019 Jul 3;14(7):e0218873. doi: 10.1371/journal.pone.0218873 (PMC6608973; doi:10.1371/journal.pone.0218873)
Supplement: S4 Table — (DOC) [file pone.0218873.s004.doc]

**Cardio Sequenzierungs-PCR**

- Probenaufbereitung mittels „QIAamp Viral RNA Mini-Kit“ von Qiagen nach entsprechender Vorschrift
- PCR-Ansatz (Master-Mix für 1 PCR-Ansatz)

RT und 1. PCR: Cardio-Univ.-F (10µM) 2 µl

Cardio-Univ.-R (10µM) 2 µl

2xSSC-ReaktionMix 12,5 µl

H_2_O 1,5 µl

BSA (1 mg/ml) 1 µl

Enzym-Mix 1 µl

20 µl des Master-Mix + 5 µl RNA-Aufbereitung.

Cyclen: 48°C 30 min

95°C 5 min

|95°C 1 min

35x |50°C 1 min

|72°C 1 min

72°C 5 min

4°C unendlich

Biometra-Zykler Programm 70 (CARDIO-1)

2. PCR: Cardio-F (10µM) 2 µl

Cardio-R (10µM) 2 µl

dNTP Mix (10mM each) 1 µl

MgCl_2_ (50mM) 2 µl

10x Platinum-Puffer 5 µl

H_2_O 32,8 µl

Platinum Taq 0,2 µl

45 µl des Master-Mix + 5 µl 1.PCR-Produkt

Cyclen: 95°C 3 min

|94°C 1 min

35x |60°C 1 min

|72°C 1 min

72°C 5 min

4°C unendlich

Biometra-Zykler Programm 71 (CARDIO-2)

- Auswertung im Gel und evtl. anschließende Sequenzierung
- Primer-Sequenzen:

CARDIO-Universal-F: GCTAATCAGAGGAAAGTCAGCATT

CARDIO-Universal-R: GACCACTTGGTTTGGAGAAGCT

CARDIO-F: CAGCATTTTCCGGCCCAGGC

CARDIO-R: ATCCACGGGGCTTTTGGCCG
