# Supplementary material for: Frequent detection of Saffold cardiovirus in adenoids
Source: PLoS One. 2019 Jul 3;14(7):e0218873. doi: 10.1371/journal.pone.0218873 (PMC6608973; doi:10.1371/journal.pone.0218873)
Supplement: S5 Table — (ZIP) [file pone.0218873.s005.zip › Cardio-Rohdaten/LC480-Cardio-Läufe.pdf]

Cardio - PCR

LightCycler® 480 Software Report

Roche

Abs Quant/Fit Points for Cardio (Abs Quant/Fit Points)

Results

Inc	Pos	Name	Type	CP	Concentration	Standard	Status
☑	A1	R1527	Unknown				
☑	A2	R2857	Unknown				
☑	A3	R2875	Unknown				
☑	A4	R2879	Unknown				
☑	A5	R2880	Unknown				
☑	A6	R2876	Unknown				
☑	A7	R2885	Unknown				
☑	A8	R2888	Unknown				
☑	A9	R2890	Unknown				
☑	A10	R2896	Unknown				
☑	A11	R2897	Unknown				
☑	A12	R2903	Unknown				
☑	B1	1G	Unknown				
☑	B2	3G	Unknown				
☑	B3	4G	Unknown	42,00			
☑	B4	5G	Unknown				
☑	B5	26G	Unknown				
☑	B6	27G neu	Unknown				
☑	B7	28G neu	Unknown				
☑	B8	29.1G	Unknown				
☑	B9	30G	Unknown	41,94			
☑	B10	31G	Unknown				
☑	B11	32G	Unknown				
☑	B12	33G	Unknown				
☑	C1	34G	Unknown				
☑	C2	35G	Unknown	38,62			
☑	C3	36G	Unknown				
☑	C4	37G	Unknown				
☑	C5	38G	Unknown				
☑	C6	39G	Unknown				
☑	C7	40G	Unknown				
☑	C8	41G	Unknown				
☑	C9	42G	Unknown				

Results

Inc	Pos	Name	Type	CP	Concentration	Standard	Status
☑	C10	43G	Unknown	32,41			
☑	C11	44G	Unknown				
☑	C12	45G	Unknown				
☑	D1	Cardio 1e-8	Unknown	28,28			
☑	D2	PK	Unknown				

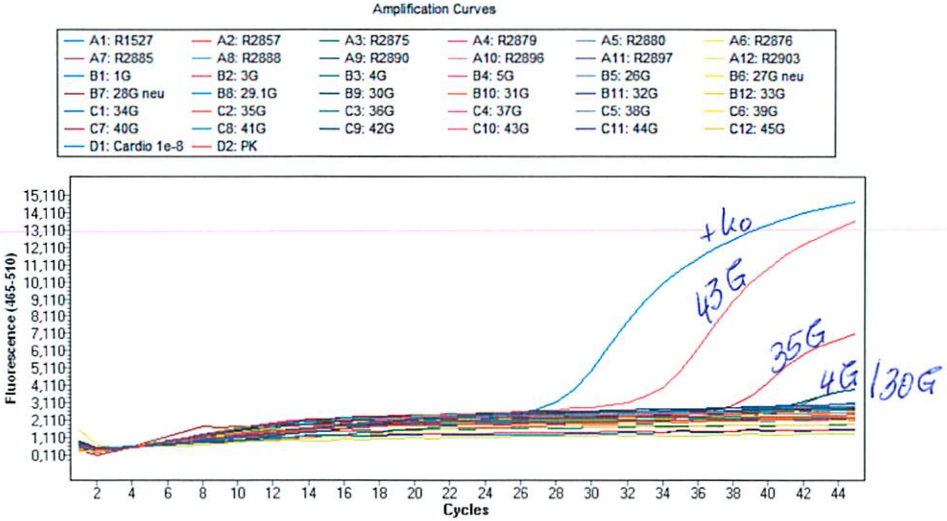

Cardio - Urina Proben

LightCycler® 480 Software

Report

Roche

Abs Quant/Fit Points for Cardio (Abs Quant/Fit Points)

Results

Inc	Pos	Name	Type	CP	Concentration	Standard	Status
☑	A1	46G	Unknown				
☑	A2	47G	Unknown				
☑	A3	48G	Unknown				
☑	A4	49G	Unknown				
☑	A5	50G	Unknown				
☑	A6	51G	Unknown				
☑	A7	52G	Unknown				
☑	A8	53G	Unknown				
☑	A9	54G	Unknown				
☑	A10	55G	Unknown				
☑	A11	56G	Unknown				
☑	A12	57G	Unknown				
☑	B1	58G	Unknown				
☑	B2	59G	Unknown				
☑	B3	60G	Unknown				
☑	B4	61G	Unknown				
☑	B5	62G	Unknown				
☑	B6	63G	Unknown				
☑	B7	64G	Unknown				
☑	B8	65.1G	Unknown				
☑	B9	66G	Unknown				
☑	B10	68G	Unknown				
☑	B11	69G	Unknown				
☑	B12	70G	Unknown				
☑	C1	71G	Unknown				
☑	C2	72G	Unknown				
☑	C3	73G	Unknown				
☑	C4	74G	Unknown				
☑	C5	75G	Unknown	29,41			
☑	C6	76G	Unknown	29,36			
☑	C7	77G	Unknown				
☑	C8	78.1G	Unknown				
☑	C9	79G	Unknown				

Results

Inc	Pos	Name	Type	CP	Concentration	Standard	Status
☑	C10	80G	Unknown				
☑	C11	81G	Unknown				
☑	C12	82.1G	Unknown				
☑	D1	83G	Unknown				
☑	D2	84G	Unknown				
☑	D3	85G	Unknown				
☑	D4	86G	Unknown				
☑	D5	87G	Unknown	34,53			
☑	D6	88G	Unknown				
☑	D7	89G	Unknown				
☑	D8	90G	Unknown				
☑	D9	91.1G	Unknown				
☑	D10	92G	Unknown				
☑	D11	93G	Unknown				
☑	D12	Cardio	Unknown	28,73			
☑	E1	95G	Unknown	34,91			
☑	E2	96G	Unknown				
☑	E3	97G	Unknown				
☑	E4	98G	Unknown				
☑	E5	99G	Unknown				
☑	E6	100G	Unknown				
☑	E7	101G	Unknown				
☑	E8	102G	Unknown	32,33			
☑	E9	103G	Unknown	42,52			
☑	E10	104G	Unknown				
☑	E11	105G	Unknown				
☑	E12	106G	Unknown				
☑	F1	107G	Unknown				
☑	F2	108G	Unknown				
☑	F3	109G	Unknown				
☑	F4	110G	Unknown				
☑	F5	111.1G	Unknown				
☑	F6	112G	Unknown				
☑	F7	113G	Unknown				
☑	F8	114G	Unknown				
☑	F9	115G	Unknown				
☑	F10	116G	Unknown				

Results

Inc	Pos	Name	Type	CP	Concentration	Standard	Status
☑	F11	117G	Unknown				
☑	F12	118G	Unknown				
☑	G1	119G	Unknown				
☑	G2	120G	Unknown				
☑	G3	121G	Unknown				
☑	G4	122G	Unknown				
☑	G5	123G	Unknown				
☑	G6	124G	Unknown				
☑	G7	125G	Unknown	39,87			
☑	G8	127G	Unknown				
☑	G9	129.2G	Unknown				
☑	G10	130G	Unknown				
☑	G11	94G	Unknown				
☑	G12	Cardio	Unknown	29,33			

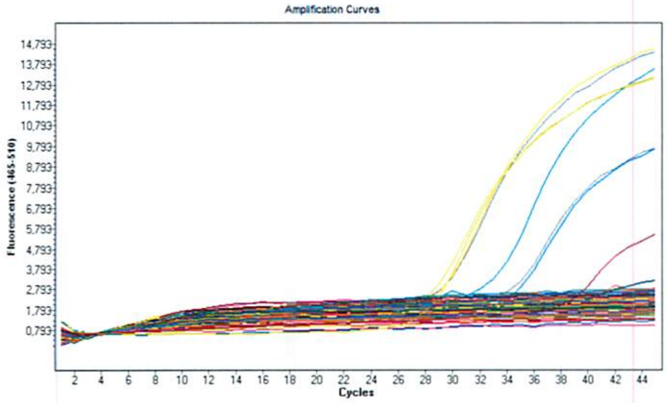

LightCycler® 480 Software
Report

Roche

Cardio (Abs Quant/2nd Derivative Max)

Results

Inc	Pos	Name	Type	CP	Concentration	Standard	Status
☑	A1	29.2G	Unknown				
☑	A2	65.2G	Unknown				
☑	A3	78.2G	Unknown				
☑	A4	91.1G	Unknown				
☑	A5	111.2G	Unknown				
☑	A6	129.1G	Unknown				
☑	A7	19A	Unknown				
☑	A8	20A	Unknown				
☑	A9	21A	Unknown				
☑	A10	22A	Unknown				
☑	A11	23A	Unknown				
☑	A12	24A	Unknown				
☑	B1	25A	Unknown				
☑	B2	26A	Unknown				
☑	B3	27A	Unknown				
☑	B4	28A	Unknown				
☑	B5	29A	Unknown				
☑	B6	29A gelb	Unknown				
☑	B7	30A	Unknown				
☑	B8	31A	Unknown				
☑	B9	32A	Unknown				
☑	B10	33A	Unknown				
☑	B11	34A	Unknown				
☑	B12	35A	Unknown				
☑	C1	36A	Unknown				
☑	C2	37A	Unknown				
☑	C3	38A	Unknown				
☑	C4	39A	Unknown				
☑	C5	40A	Unknown				
☑	C6	41A	Unknown				
☑	C7	42A	Unknown				
☑	C8	43A	Unknown				
☑	C9	44A	Unknown				

Results

Inc	Pos	Name	Type	CP	Concentration	Standard	Status
☑	C10	45A	Unknown				
☑	C11	46A	Unknown				
☑	C12	47A	Unknown				
☑	D1	81A	Unknown				
☑	D2	82A	Unknown				
☑	D3	83A	Unknown				
☑	D4	84A	Unknown				
☑	D5	85A	Unknown				
☑	D6	86A	Unknown				
☑	D7	87A	Unknown	33,17			
☑	D8	89A	Unknown				
☑	D9	90A	Unknown				
☑	D10	91A	Unknown				
☑	D11	92A	Unknown				
☑	D12	93A	Unknown				
☑	E1	94A	Unknown				
☑	E2	95A	Unknown				
☑	E3	96A	Unknown				
☑	E4	97A	Unknown				
☑	E5	98A	Unknown				
☑	E6	99A	Unknown				
☑	E7	100A	Unknown				
☑	E8	101A	Unknown				
☑	E9	102A	Unknown				
☑	E10	103A	Unknown				
☑	E11	104A	Unknown				
☑	E12	105A	Unknown				
☑	F1	106A	Unknown				
☑	F2	107A	Unknown				
☑	F3	108A	Unknown				
☑	F4	109A	Unknown				
☑	F5	111A	Unknown				
☑	F6	112A	Unknown				
☑	F7	113A	Unknown				
☑	F8	114A	Unknown				
☑	F9	115A	Unknown				
☑	F10	116A	Unknown				

Results

Inc	Pos	Name	Type	CP	Concentration	Standard	Status
☑	F11	117A	Unknown				
☑	F12	118A	Unknown				
☑	G1	PK	Unknown				
☑	G2	Cardio	Unknown	27,06			
☑	G3	119A	Unknown				
☑	G4	120A	Unknown				
☑	G5	121A	Unknown				
☑	G6	122A	Unknown				
☑	G7	123A	Unknown				
☑	G8	124A	Unknown				
☑	G9	125A	Unknown				
☑	G10	126A	Unknown				
☑	G11	127A	Unknown				
☑	G12	128A	Unknown				
☑	H1	129A	Unknown				
☑	H2	131A	Unknown				
☑	H3	132A	Unknown				
☑	H4	130A	Unknown				

Amplification Curves

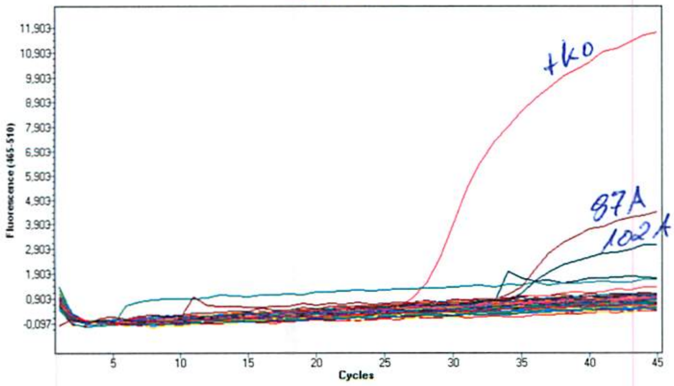

87A } entspr.
102A } Gewebe-
proben
Cardio pos.
